# Benefits of Resistance Training in Early and Late Stages of Frailty and Sarcopenia: A Systematic Review and Meta-Analysis of Randomized Controlled Studies

**DOI:** 10.3390/jcm10081630

**Published:** 2021-04-12

**Authors:** Karolina Talar, Alejandro Hernández-Belmonte, Tomas Vetrovsky, Michal Steffl, Ewa Kałamacka, Javier Courel-Ibáñez

**Affiliations:** 1 Faculty of Motor Rehabilitation, University of Physical Education, 31-571 Krakow, Poland; karotalar@gmail.com (K.T.); ewa.kalamacka@awf.krakow.pl (E.K.); 2 Human Performance and Sports Science Laboratory, Faculty of Sport Sciences, University of Murcia, 30730 Murcia, Spain; alejandro.hernandez7@um.es; 3 Faculty of Physical Education and Sport, Charles University, 16252 Prague, Czech Republic; steffl@ftvs.cuni.cz

**Keywords:** aging, older adults, muscle mass, weakness, exercise, physical performance

## Abstract

Sarcopenia and frailty are age-related syndromes with negative effects on the quality of life of older people and on public health costs. Although extensive research has been carried out on the effects of physical exercise and physical syndromes, there is a knowledge gap when it comes to the effect of resistance training on muscular strength, physical performance, and body composition at early (prevention) and late (treatment) stages in both syndromes combined. We conducted this systematic review and meta-analysis (CRD42019138253) to gather the evidence of randomized controlled trials examining the effects of resistance training programs lasting ≥8 weeks on strength, physical function, and body composition of adults ≥65 years old diagnosed with pre-sarcopenia, sarcopenia, pre-frailty, or frailty. A search from the earliest record up to and including December 2020 was carried out using the PubMed, Scopus, Web of Science, and Cochrane Library databases. A total of 25 studies (*n* = 2267 participants) were included. Meta-analysis showed significant changes in favour of resistance training for handgrip (ES = 0.51, *p* = 0.001) and lower-limb strength (ES = 0.93, *p* < 0.001), agility (ES = 0.78, *p* = 0.003), gait speed (ES = 0.75, *p* < 0.001), postural stability (ES = 0.68, *p* = 0.007), functional performance (ES = 0.76, *p* < 0.001), fat mass (ES = 0.41, *p* = 0.001), and muscle mass (ES = 0.29, *p* = 0.002). Resistance training during early stages had positive effects in all variables during early stages (ES > 0.12), being particularly effective in improving gait speed (ES = 0.63, *p* = 0.016) and functional strength (ES = 0.53, *p* = 0.011). Based on these results, resistance training should be considered as a highly effective preventive strategy to delay and attenuate the negative effects of sarcopenia and frailty in both early and late stages.

## 1. Introduction

Life expectancy is the highest to date, and world aging has increased at a staggering rate [1]. Despite the fact that people live longer than ever, human aging produces various syndromes that reduce their quality of life, contribute to their dependence, and increase public care costs [2]. Prominent among these syndromes are sarcopenia and frailty. A recent estimate from 28 European countries suggests increments of 60–70% in the prevalence of sarcopenia by 2045, resulting in 12.9 to 22.3% of people over 65 years old being affected [3]. Sarcopenia is generated by a severe loss of muscle mass as a consequence of diverse factors such as nutritional status, physical activity, genetic heritability, or hormonal changes [4]. This fact, together with a decline in the tendon proprieties [5] and neural patterns [6], results in a loss of muscular strength and mobility (i.e., functional status). Frailty, for its part, is an age-associated medical syndrome that embodies a high risk for falls, disability, hospitalization, and mortality among older adults [7]. Frailty has been shown to increase health costs by up to ~5 times [8]. Therefore, these age-related physical syndromes require the implementation of treatment aimed to reduce the public health costs, but above all, to attenuate the loss of quality of life among older adults suffering from them. Frailty can lead to common healthcare issues, such as decrease of strength, immobility, falls, undernutrition, incontinence, depression and anxiety [9]. In addition to healthy lifestyle behaviours, frailty may be prevented and even reversed with proper exercise training [10,11,12]. Among frail older adults, exercise is particularly important to maintain physical functioning and self-autonomy, reducing the risk of falls, acute hospital and care home admission [13,14].

On this matter, supervised exercise is proposed as an effective strategy to treat sarcopenia and physical frailty [12,14]. Chiefly, resistance training interventions might be particularly beneficial to delay and reduce the causes (e.g., loss of muscle mass) and consequences (e.g., loss of muscular strength or functionality) that both syndromes usually produce, even at early stages [15,16]. Resistance training is defined as a strength training exercise with the use of progressive overload in which the muscles create the force against external load [17]. Moreover, resistance training is the most effective exercise type intervention compared to endurance training or the whole-body vibration training and can improve physical function and physical performance in older adults [18]. Resistance training as an essential component of a complete exercise program complements the commonly known positive effects of aerobic training on health and physical capacities [19,20,21]. Previous research has been carried out on the effects of physical exercise interventions on frailty and/or sarcopenia [22,23], and of resistance training on frailty [24] or sarcopenia [25]. However, there is a knowledge gap when it comes to the effect of resistance training on muscular strength, physical performance, and body composition at early (prevention) and late (treatment) stages in both syndromes combined. Besides, little is known about the potential effect of resistance training as a preventive strategy to reduce the occurrence of these syndromes [25].

Because of its potential, research examining resistance training effects on age-related physical syndromes is on the rise. Therefore, an update of the state of the art is required. This research aimed to systematically review the scientific evidence examining the effect of resistance training on muscular strength, physical function, and body composition of older adults diagnosed with pre-sarcopenia, sarcopenia, pre-frailty, or frailty. Moreover, to address this issue comprehensively, a meta-analysis was conducted to synthesize the outcomes of comparative studies. Based on the available literature, it was expected that older adults diagnosed with frailty or sarcopenia at both early and late stages would demonstrate improvement in handgrip strength, lower-limb strength, muscle mass, and functional performance after eight or more weeks of resistance training.

## 2. Materials and Methods

### 2.1. Registration of Systematic Review Protocol

The protocol of this investigation was pre-registered in the PROSPERO database (CRD42019138253). The present systematic review and meta-analysis were conducted according to the Preferred Reporting Items for Systematic Reviews and Meta-Analyses (PRISMA) statement [26].

### 2.2. Identification and Selection of Studies

A search from the earliest record up to and including December 2020 was carried out using the following electronic databases: Medline, Scopus, Web of Science, and Cochrane Library. The search strategy combined terms related to the population (e.g., sarcopenia, frailty) and intervention (e.g., resistance training, strength training). Table A1 shows the full Boolean search syntax used in PubMed. The PubMed syntax was then adapted for the search in the Web of Science and Scopus. Additionally, the article’s reference lists were scanned to identify additional studies for inclusion in the present review. The titles and abstracts of the retrieved articles were individually evaluated for eligibility. Potentially eligible articles were retrieved for full text evaluation. If any disagreement occurred, a consensus meeting was held between all the reviewers to reach an agreement upon inclusion of the publication.

### 2.3. Eligibility Criteria

The eligibility criteria were: (1) participants included older individuals (≥65 years of age) with pre-frailty, frailty, pre-sarcopenia or sarcopenia but without comorbid conditions (e.g., diabetes, cancer, stroke, dementia, depression); (2) resistance training intervention lasted ≥ 8 weeks as this is the recommended minimum intervention duration to increase muscle strength and treat sarcopenia [27]. Moreover, muscle hypertrophy is observed after 8 weeks with longer training periods supporting lasting effects [28]; (3) at least one outcome of interest (muscular strength, body composition, gait speed, balance, agility) was reported before and after the training intervention; (4) randomized controlled trial as study design; (5) manuscript published in English (dissertations and conference proceedings were excluded). Studies including other interventions as controls (supplementation, home-based exercise, educational programs, or combined interventions) were included.

### 2.4. Data Extraction

The following variables from the included studies were extracted independently by two authors (KT and MS): (1) characteristics of the study (year of publication, geographical area) and the sample (size, gender, and age); (2) description of the program conducted by the training and control group; (3) main outcomes of interest; and (4) overall effect of the outcome of interest. For quantitative analyses (meta-analyses), authors collected the group size and mean differences of the outcomes of interest with a 95% confidence interval (CI) or standard deviation (SD) for both groups (intervention and control). All data were tabulated in an Excel spreadsheet (Microsoft Corporation, Redmond, WA, USA) predesigned for this review. Coding sheets were cross-checked between authors, while discussion and consensus resolved any discrepancies.

### 2.5. Methodological Quality

As described in our PROSPERO protocol, we initially intended to use the GRADE scale as a widely recommended system for observational studies and randomized controlled trials [29]. However, subsequent to the protocol registration, we decided to use the Physiotherapy Evidence Database (PEDro) scale to assess the methodological quality of included studies [30]. PEDro provides an assessment of the quality of randomized controlled trials, especially in evidence-based physical-therapy [31]. The PEDro scale has demonstrated reliability with score range from 0 to 11, where scores ≤ 3 represent poor study quality, scores of 4–5 indicate fair quality, and scores ≥ 6 represent good to excellent quality [32]. This change to the protocol has been registered in PROSPERO.

### 2.6. Statistical Analysis

The effect sizes (ES) were calculated as the standardized mean differences between the resistance training group and the control group. Sub-group analyses were conducted to examine ES for early stages of sarcopenia and frailty. As traditional meta-regression methods do not allow for multiple dependent outcomes from the same study to be included in one analysis, we used a meta-analytic method for dealing with dependent effect sizes named robust variance estimation (RVE). RVE is a form of random-effects meta-regression for multilevel data structures, which allows for multiple effect sizes from the same study to be included in a meta-analysis, even when information on the covariance of these effect sizes is unavailable. Instead, RVE estimates the variance of meta-regression coefficient estimates using the observed residuals. It does not require distributional assumptions and does not make any requirements on the weights [33,34]. A study was used as the clustering variable to account for correlated effects within studies. Observations were weighted by the inverse of the sampling variance. A sensitivity analysis using alternative correlational values to calculate the standard error revealed that the choice of correlational value did not impact the overall results of the meta-analysis. I^2^ was used to evaluate between-study heterogeneity. Values of I^2^ more than 25%, 50%, and 75% were selected to reflect low, moderate, and high heterogeneity, respectively [35]. All analyses were performed using packages robumeta (version 2.0) and metafor (version 2.0-0) in R version 3.4.4 (The R Foundation for Statistical Computing, Vienna, Austria).

## 3. Results

### 3.1. Study Selection

**Figure 1:**
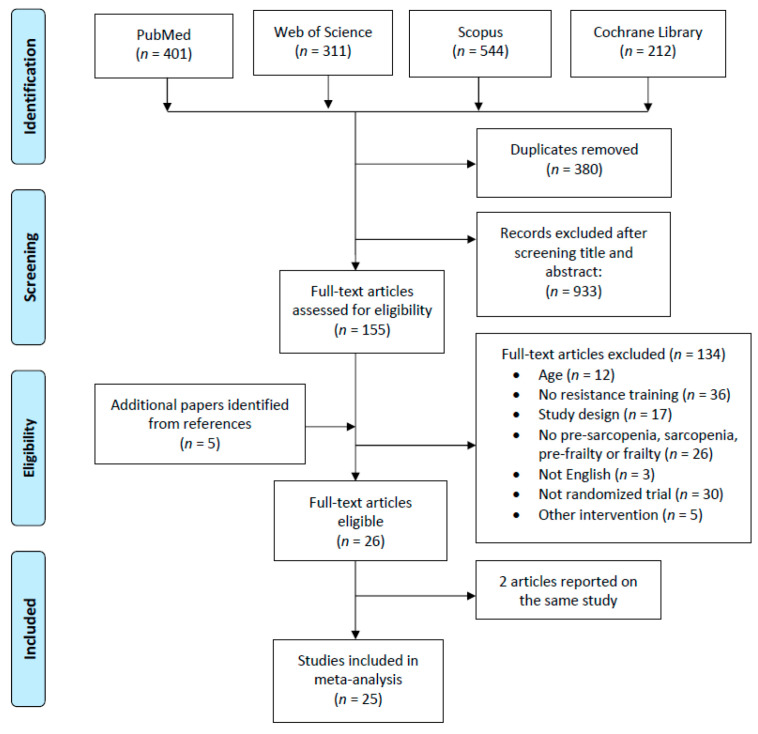
Study retrieval process according to the Preferred Reporting Items for Systematic Reviews and Meta-Analyses (PRISMA) statements.

The database search yielded 1468 articles. Of those, 155 full texts were retrieved, and 26 deemed eligible [37,38,39,40,42,43,44,45,46,48,49,50,51,52,53,54,55,56,57,58,59,60,61,]. As one study reported its results in two separate articles, 25 studies were included in this review, as shown in the PRISMA flow diagram (Figure 1).

### 3.2. Methodological Quality

The methodological quality of the studies is detailed in Table 1. Since all the studies obtained the predefined minimum score of 6 points, they were all included in the qualitative and quantitative syntheses. The minimum, maximum, and mean scores of the quality analysis were 6, 11, and 8.76 (±1.26) points, respectively.

### 3.3. Study Characteristics

In total, there were 2267 participants (1484 women). The mean age ranged from 62 to 98 years. The mean duration of resistance training programs was approximately 23 weeks (range 10–48 weeks), and the most common training frequency was 2–3 times per week (in 21 studies). Table 2 and Table 3 show the characteristics and the overall effect of the 25 studies included in the review. Table A2 presents the diagnostic criteria for frailty or sarcopenia with the prevalence of participants for each study.

### 3.4. Muscular Strength

**Figure 2:**
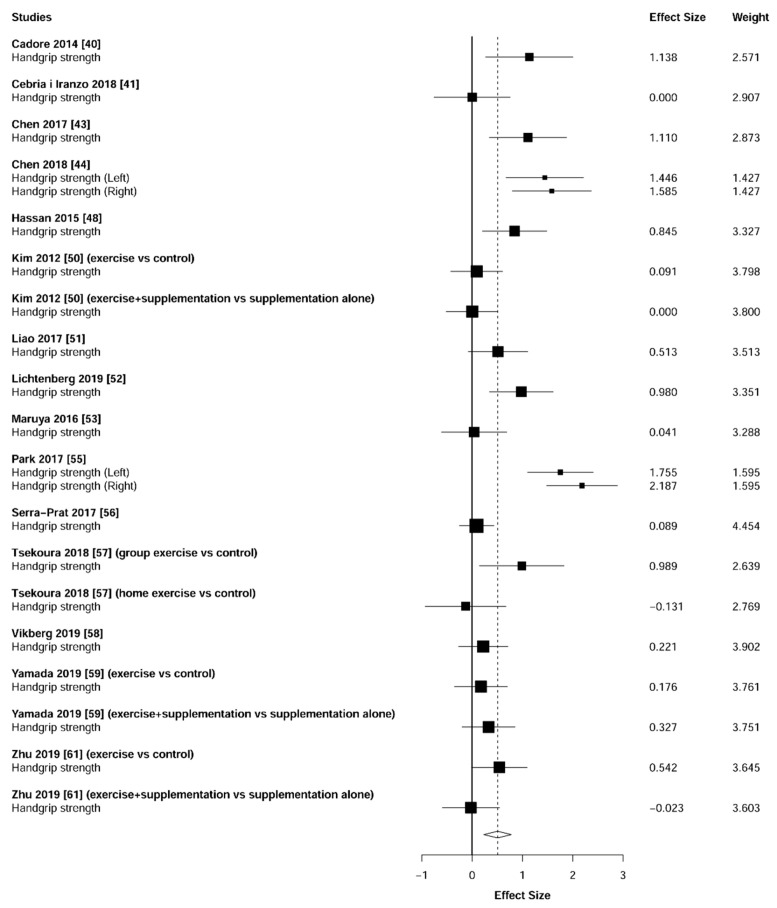
Forest plot showing the comparative effect of resistance training vs. control group on the handgrip. Effect sizes greater than zero favor resistance training.

**Figure 3:**
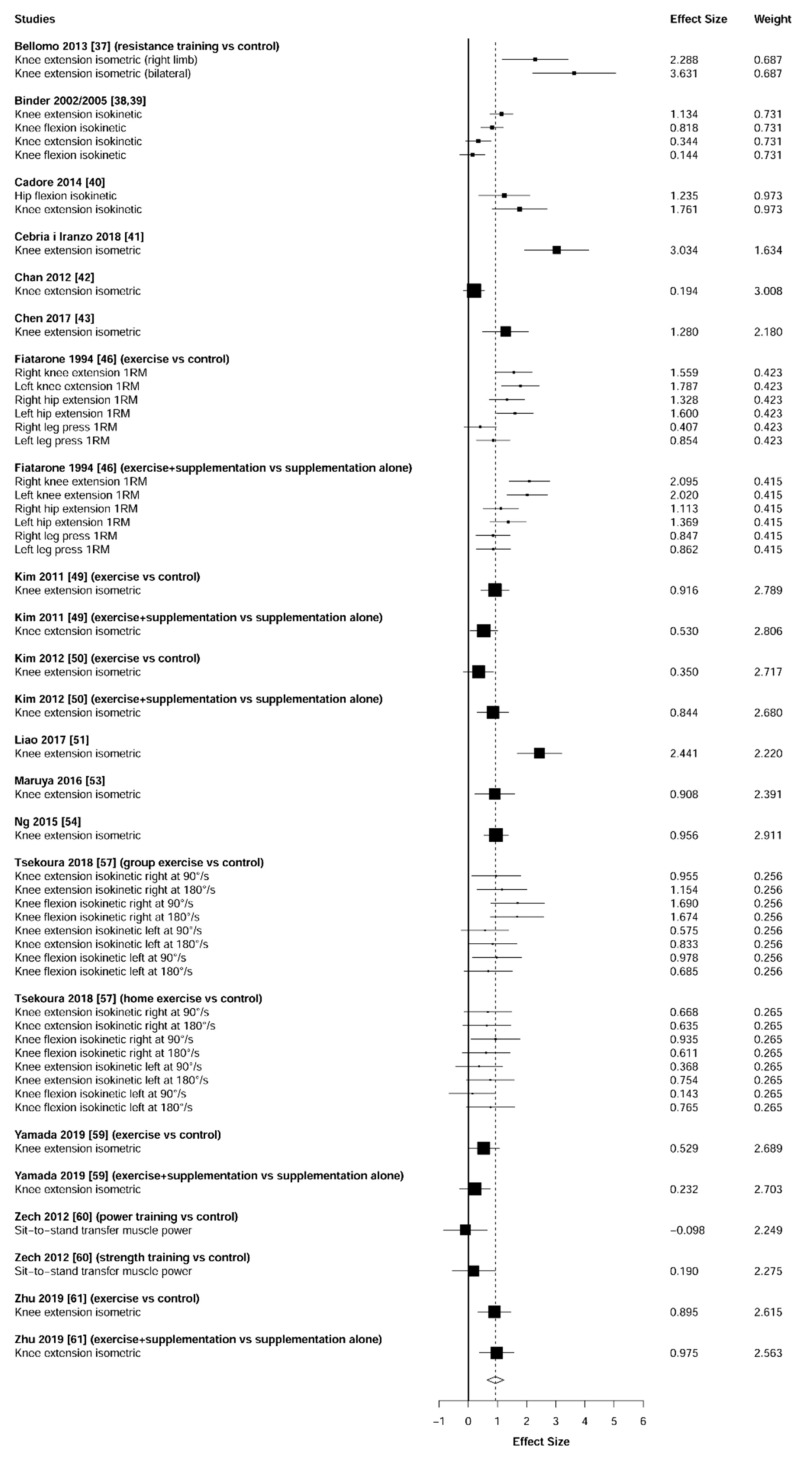
Forest plot showing the comparative effect of resistance training vs. control group on lower-limb strength. Effect sizes greater than zero favor resistance training.

**Figure 4:**
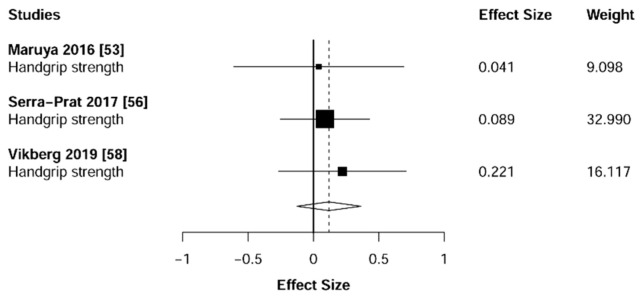
Subgroup analysis for early stages (pre-frailty or pre-sarcopenia). Forest plot showing the comparative effect of resistance training vs. control group on the handgrip. Effect sizes greater than zero favor resistance training.

**Figure 5:**
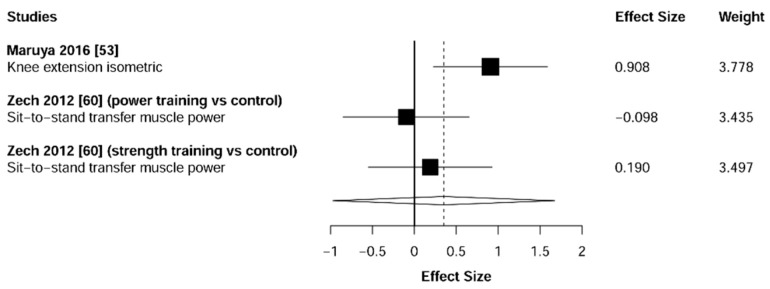
Subgroup analysis for early stages (pre-frailty or pre-sarcopenia). Forest plot showing the comparative effect of resistance training vs. control group on lower-limb strength. Effect sizes greater than zero favor resistance training.

Meta-analysis showed significant changes in handgrip (ES = 0.51 [95% CI: 0.23 to 0.78], *p* = 0.001, Figure 2) and lower-limb strength (ES = 0.93 [95% CI: 0.64 to 1.22], *p* < 0.001, Figure 3) in favor of the resistance training group. Heterogeneity of the results around these outcomes was moderate for the handgrip (I^2^ = 68%) and high for the lower-limb strength (I^2^ = 77%). Sub-group analyses for early stages yielded positive but non-significant effects in handgrip (ES = 0.12 [95% CI: -0.13 to 0.36], I^2^ = 0%, *p =* 0.146, Figure 4) and lower-limb strength (ES = 0.35 [95% CI: −0.97 to 1.67], I^2^ = 52%, *p* = 0.372, Figure 5).

### 3.5. Physical Function

**Figure 6:**
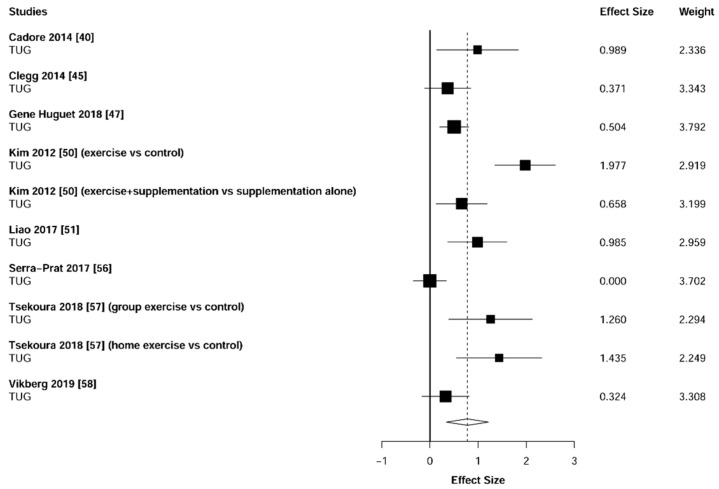
Forest plot showing the comparative effect of resistance training vs. control group on agility. Effect sizes greater than zero favor resistance training. TUG: Timed Up & Go test.

**Figure 7:**
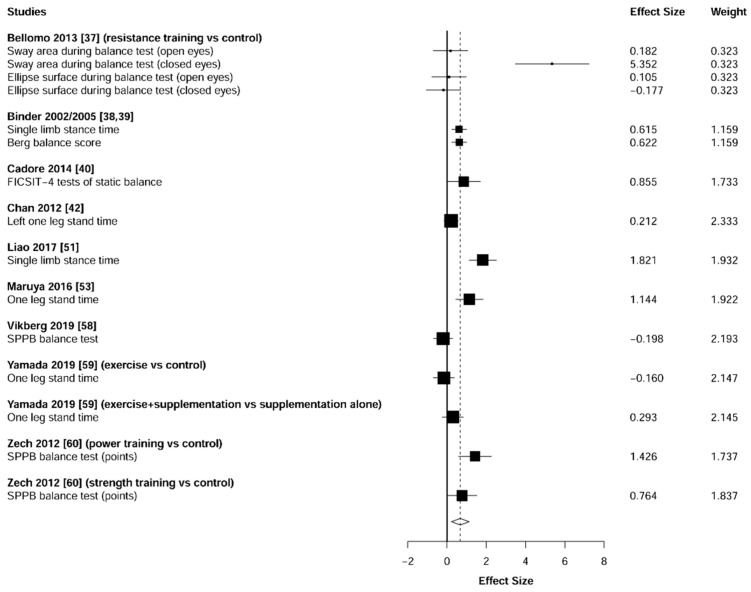
Forest plot showing the comparative effect of resistance training vs. control group on balance. Effect sizes greater than zero favor resistance training.

**Figure 8:**
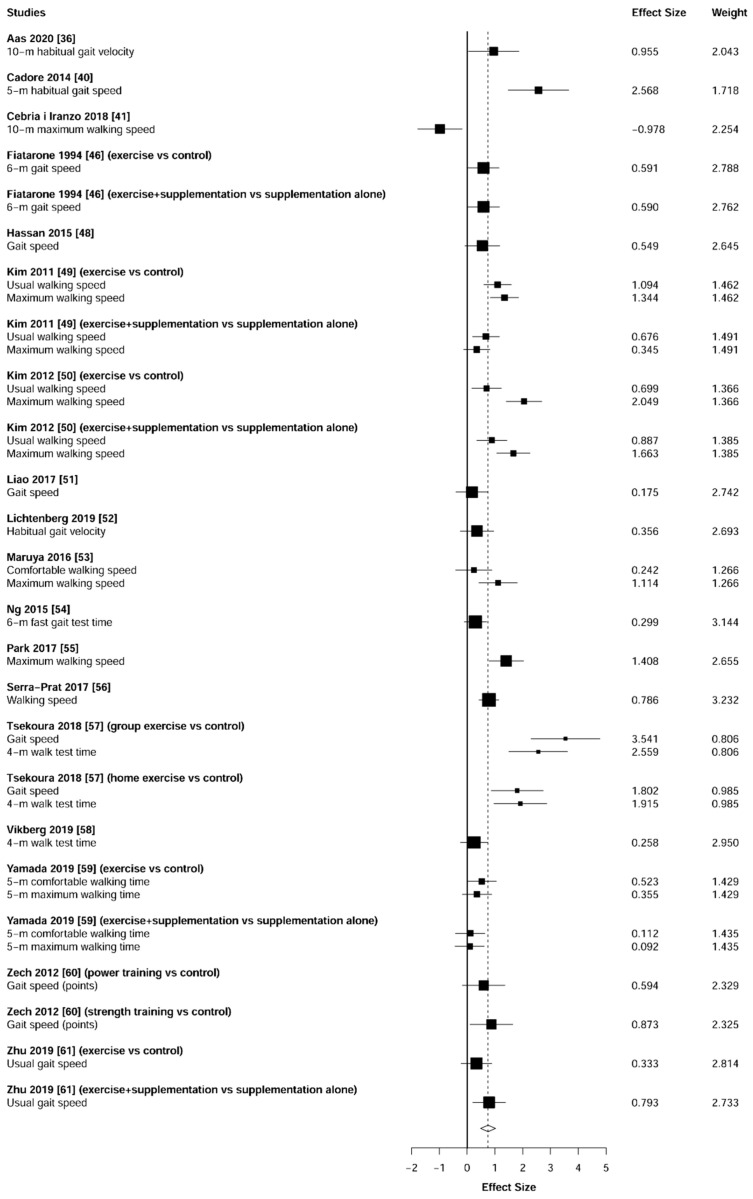
Forest plot showing the comparative effect of resistance training vs. control group on gait speed. Effect sizes greater than zero favor resistance training.

**Figure 9:**
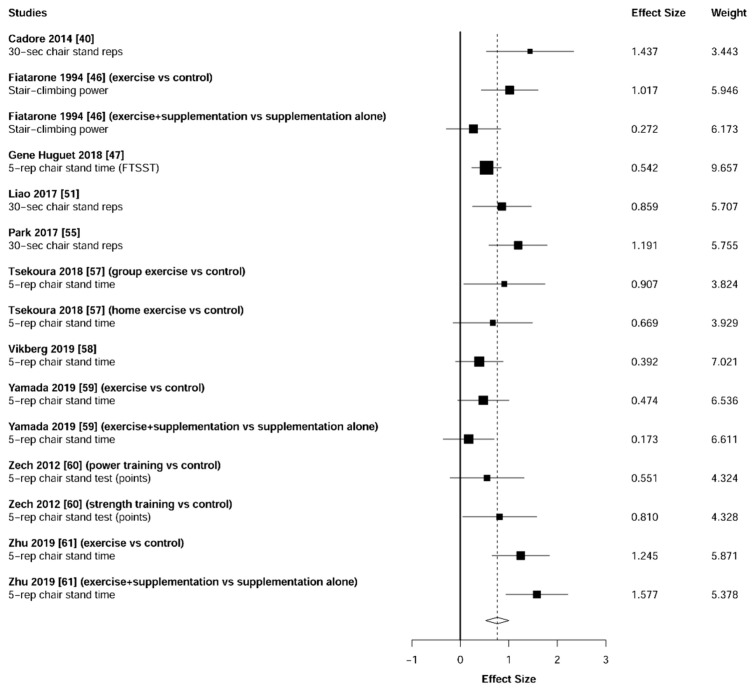
Forest plot showing the comparative effect of resistance training vs. control group on functional strength. Effect sizes greater than zero favor resistance training.

**Figure 10:**
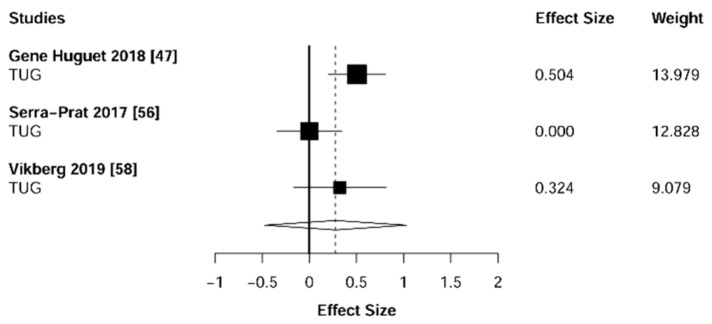
Subgroup analysis for early stages (pre-frailty or pre-sarcopenia). Forest plot showing the comparative effect of resistance training vs. control group on agility. Effect sizes greater than zero favor resistance training. TUG: Timed Up & Go test.

**Figure 11:**
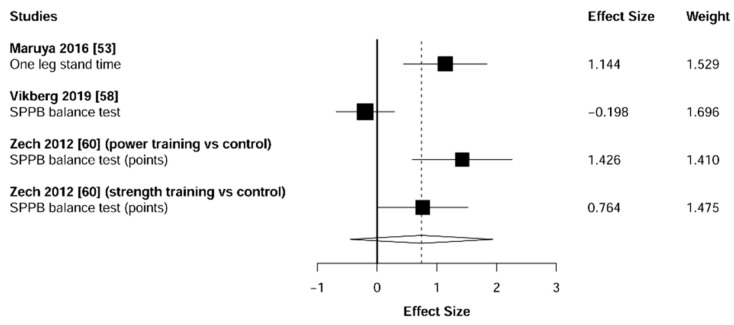
Subgroup analysis for early stages (pre-frailty or pre-sarcopenia). Forest plot showing the comparative effect of resistance training vs. control group on balance. Effect sizes greater than zero favor resistance training.

**Figure 12:**
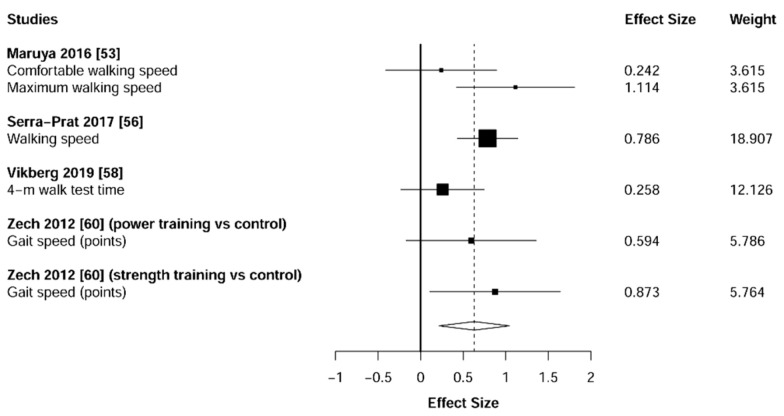
Subgroup analysis for early stages (pre-frailty or pre-sarcopenia). Forest plot showing the comparative effect of resistance training vs. control group on gait speed. Effect sizes greater than zero favor resistance training.

**Figure 13:**
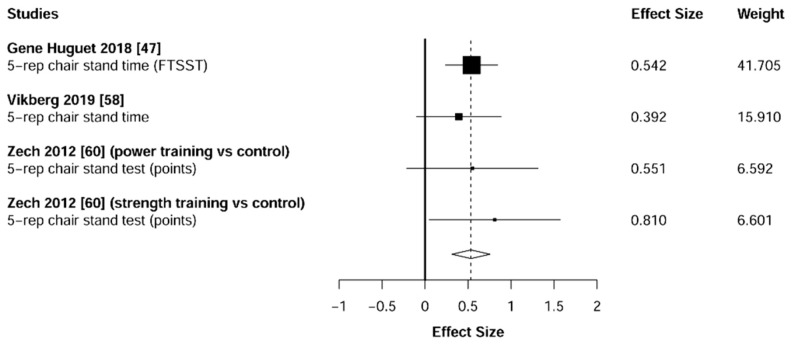
Subgroup analysis for early stages (pre-frailty or pre-sarcopenia). Forest plot showing the comparative effect of resistance training vs. control group on functional strength. Effect sizes greater than zero favor resistance training.

Meta-analysis showed significant changes in favor of the resistance training group for the agility (ES = 0.78 [95% CI: 0.34 to 1.22], *p* = 0.003, Figure 6), balance (ES = 0.68 [95% CI: 0.23 to 1.13], *p* = 0.007, Figure 7), gait speed (ES = 0.75 [95% CI: 0.49 to 1.02], *p* < 0.001, Figure 8), and functional strength (ES = 0.76 [95% CI: 0.52 to 1.00], *p* < 0.001, Figure 9). Heterogeneity of the results around these outcomes was low for the functional strength (I^2^ = 48%), and high for the gait speed (I^2^ = 76%), postural stability (I^2^ = 82%), and agility (I^2^ = 78%). Sub-group analyses for early stages yielded positive but non-significant effects in agility (ES = 0.28 [95% CI: −0.47 to 1.03], I^2^ = 58%, *p =* 0.244, Figure 10) and balance (ES = 0.75 [95% CI: -0.45 to 1.94], I^2^ = 82%, *p =* 0.141, Figure 11), while benefits in gait speed (ES = 0.63 [95% CI: 0.22 to 1.04], I^2^ = 18%, *p* = 0.016, Figure 12), and functional strength (ES = 0.53 [95% CI: 0.31 to 0.76], I^2^ = 0%, *p =* 0.011, Figure 13) remained significant during early stages.

### 3.6. Body Composition

**Figure 14:**
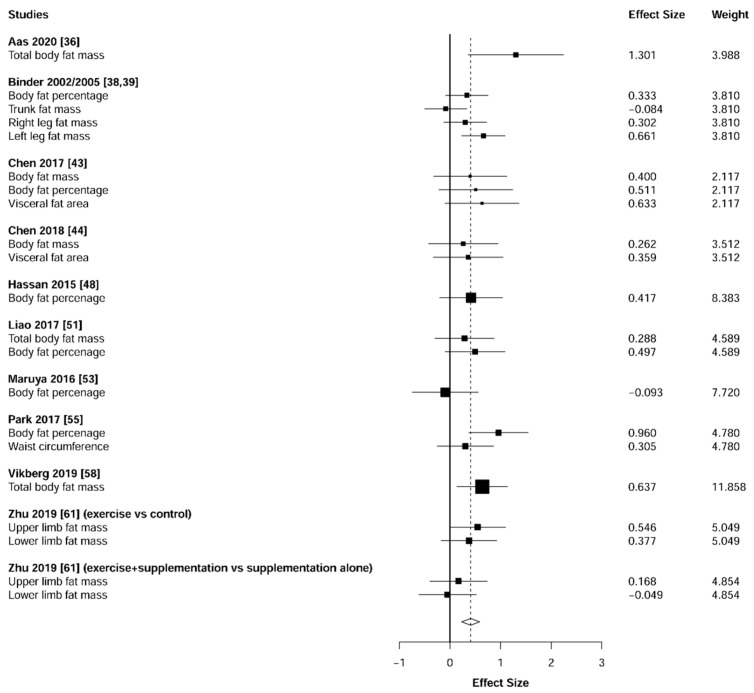
Forest plot showing the comparative effect of resistance training vs. control group on fat mass. Effect sizes greater than zero favor resistance training.

**Figure 15:**
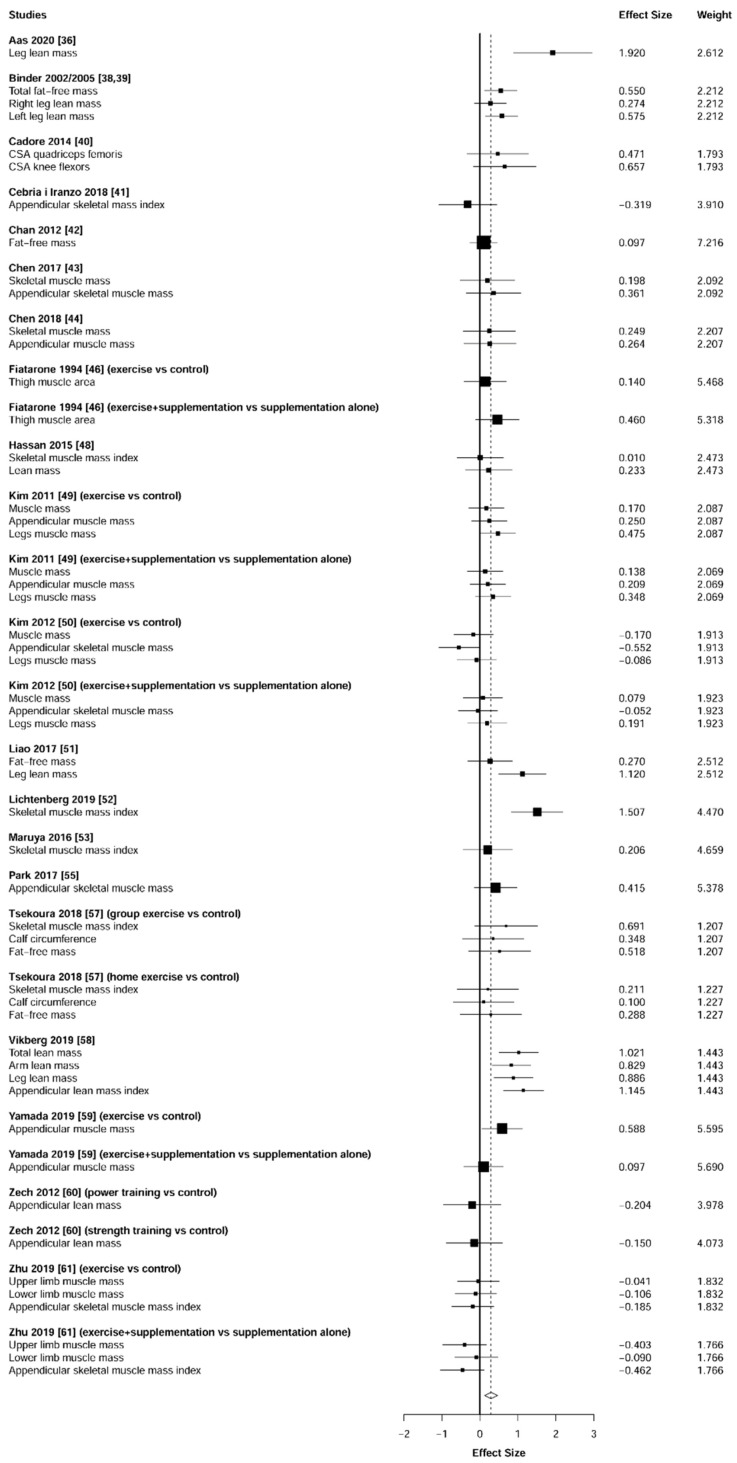
Forest plot showing the comparative effect of resistance training vs. control group on muscle mass. Effect sizes greater than zero favor resistance training.

**Figure 16:**
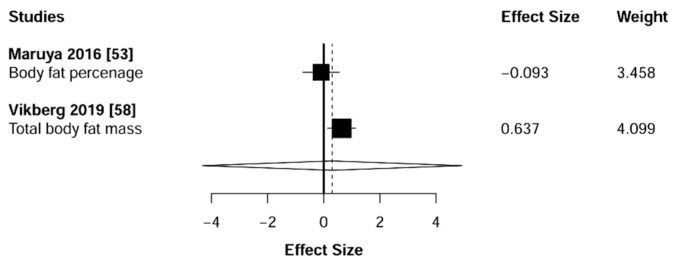
Subgroup analysis for early stages (pre-frailty or pre-sarcopenia). Forest plot showing the comparative effect of resistance training vs. control group on fat mass. Effect sizes greater than zero favor resistance training.

**Figure 17:**
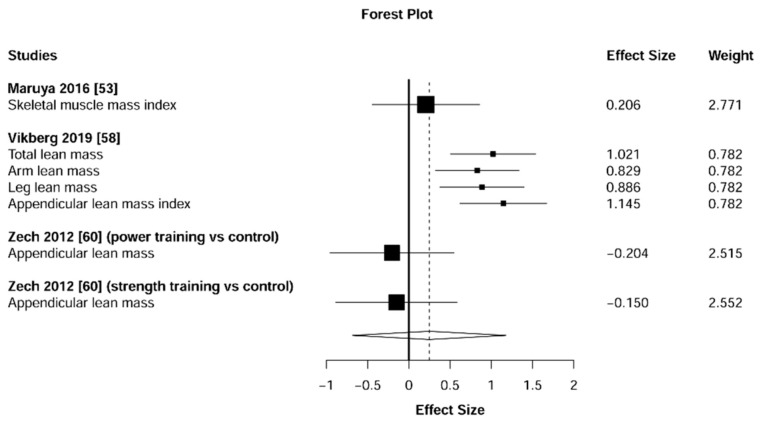
Subgroup analysis for early stages (pre-frailty or pre-sarcopenia). Forest plot showing the comparative effect of resistance training vs. control group on muscle mass. Effect sizes greater than zero favor resistance training.

Meta-analysis showed significant changes in fat mass (ES = 0.41 [95% CI: 0.23 to 0.59], *p* = 0.001, Figure 14) and muscle mass (ES = 0.29 [95% CI: 0.12 to 0.46], *p* = 0.002, Figure 15) and in favor of the resistance training group. Heterogeneity of the results around the body composition outcomes was very low for the fat mass (I^2^ = 18%) and moderate for the muscle mass (I^2^ = 54%). Sub-group analyses for early stages yielded positive but non-significant effects in fat mass (ES = 0.30 [95% CI: −4.32 to 4.92], I^2^ = 67%, *p =* 0.558, Figure 16) and muscle mass (ES = 0.25 [95% CI: −0.68 to 1.18], I^2^ = 69%, *p =* 0.458, Figure 17).

## 4. Discussion

This systematic review found that resistance training is a highly effective strategy to improve muscular strength, physical function, and body composition parameters in older adults with pre-frailty, frailty, pre-sarcopenia, or sarcopenia. Besides, resistance training during early stages had positive effects in all variables, being particularly effective in improving physical function. These findings reinforce the use of strength training interventions to delay and attenuate negative effects related to both physical syndromes.

### 4.1. Muscular Strength

Muscular strength is considered the primary determinant of sarcopenia [62]. To date, the handgrip evaluation represents the most common test used to measure this physical capacity [63] due to its high affordability, portability, simplicity, and test-retest repeatability [64]. Nevertheless, to obtain an overall indicator of strength, some investigations have suggested the need to complement this test with specific evaluations of the lower-limb muscles (e.g., isometric knee extension) [65,66,67]. The present study found that individuals suffering from (pre-) sarcopenia or (pre-) frailty significantly improved both handgrip (ES = 0.51, *p* = 0.001) and lower-limb (ES = 0.93, *p* < 0.001) strength after a training intervention based on resistance exercises. Indeed, except for one study in each analysis (handgrip [57] and lower-limb [60]), all investigations reported effects in favor of the resistance training group (Figure 2 and Figure 3). Moreover, specifically to lower-limb strength, our results revealed that these strength enhancements were detected both isometrically [37,43,49,51,61,] and dynamically [38,39,40,46].

### 4.2. Physical Function

We found that all of the analyzed functional capacities were significantly improved by the implementation of a resistance training intervention (ES from 0.68 to 0.78). With the exception of one study for the agility [56] and gait [] tasks (Figure 6 and Figure 8), and two investigations for the balance task [58,59] (Figure 7), all studies found effects in favor of the resistance training group. Furthermore, all investigations reported superior effects for the resistance training group in relation to functional tasks (Figure 9). These findings could be strongly related to the significant lower-limb strength gains (Figure 3). Since the lower-limb muscles (e.g., knee extensors) are mainly responsible for actions such as chair rising or walking [68,69,70], the increment of strength in these structures could have been positively transferred into the physical function. In turn, these improvements in physical function can potentially reduce the dependency situation of older adults, thus increasing their quality of life [71] and decreasing the public health costs [72,73].

### 4.3. Body Composition

Our results revealed a positive effect of resistance training on the reduction of fat mass (ES = 0.41, *p* = 0.001, Figure 8) and increases in muscle mass (ES = 0.29, *p* = 0.002, Figure 9). Since the muscle mass can explain approximately 60–70% of strength capacity [74], these muscle mass enhancements are strongly related to the strength gains described above (Figure 2 and Figure 3). Similarly, the increases in muscle mass could have generated the decreases in fat mass as a result of the rise in the energy expenditure of the individuals [75,76]. Together, these positive changes in body composition parameters could reduce the risk of other common diseases in older adults, such as metabolic syndrome [77,78,79]. 

Generally, exercise interventions can decrease the prevalence of frailty and sarcopenia and are also effective in reducing the severity of these syndromes [12]. Our results are consistent with previous studies supporting that resistance training is beneficial for the muscular strength and physical function in older adults with frailty or sarcopenia [10,22,24], but they do not combine both syndromes (sarcopenia and frailty) and if they do, they examine the effect of exercise overall [23]. These new results support evidence that resistance training is the most effective exercise type of intervention to improve muscle strength and physical performance in older people compared to endurance training or whole-body vibration training [18]. More specifically, it seems preferable to perform multi-component exercise programs combining a power-oriented resistance training regime with endurance and balance exercises [11,80]. 

Great emphasis should also be placed on the issue of financial sustainability of healthcare. It has been observed that frailty and sarcopenia lead to the increase of public health costs [81,82,83,84,85]. According to Bock et al., 2016 the mean total 3-month costs of frail participants in Saarland, Germany were € 3659 and non-frail older adults € 642, thus more than 80% of costs could be easily saved [8]. For this reason, preventing, postponing or even reducing frailty could potentially decrease total healthcare costs in many countries.

## 5. Limitations

This study is not exempt from limitations. Firstly, except for the functional strength and fat mass, most of the meta-analyses indicated moderate to high levels of heterogeneity. This fact could be explained mainly by the different variables included in the quantitative analysis (i.e., clinical diversity), as well as by the different methodologies (e.g., volume, intensity, exercise, program duration) used in each study (i.e., methodological diversity). Secondly, although the mean duration of training interventions included in the present review (~20 weeks) allows us to suggest that resistance training is an effective short/medium-term strategy, more evidence including longer resistance training programs is needed to confirm the long-term benefits, in particular, whether they are effective for reducing prevalence of sarcopenia and frailty. Thirdly, future systematic reviews are encouraged to examine the effects of resistance training on other physiological parameters, such as the neural drive, muscle architecture, or tendon proprieties, among individuals with pre-sarcopenia, sarcopenia, pre-frailty, or frailty.

## 6. Conclusions

Based on these results, resistance training should be considered as a highly effective preventive strategy to delay and attenuate the negative effects of sarcopenia and frailty in both early and late stages.

## Figures and Tables

**Table 1:** Methodological quality assessment of Included randomized controlled trials (RCTs): Physiotherapy Evidence Database (PEDro) Scale.

Study	1	2	3	4	5	6	7	8	9	10	11	Total
Aas et al. 2019 [36]	1	1	1	1	0	0	0	1	1	1	1	8
Bellomo et al. 2013 [37]	1	1	1	1	0	0	1	1	1	1	1	9
Binder et al. 2002/2005 [38,39]	1	1	1	1	0	1	1	1	1	1	1	10
Cadore et al. 2014 [40]	1	1	1	1	0	1	1	1	1	1	1	10
Cebria i Iranzo et al. 2018 [41]	1	1	1	1	0	1	1	1	1	1	1	10
Chan et al. 2012 [42]	1	1	1	1	0	0	1	1	1	1	1	9
Chen et al. 2017 [43]	1	1	1	1	0	1	0	0	1	1	1	8
Chen et al. 2018 [44]	1	1	0	1	0	0	0	1	1	1	1	7
Clegg et al. 2014 [45]	1	1	1	1	0	0	1	0	1	1	1	9
Fiatarone et al. 1994 [46]	1	1	0	1	0	0	0	1	1	1	1	7
Gene Huguet et al. 2018 [47]	1	1	0	1	0	0	0	1	1	1	1	7
Hassan et al. 2015 [48]	1	1	1	1	1	0	0	1	1	1	1	9
Kim et al. 2011 [49]	1	1	1	1	0	1	1	1	1	1	1	10
Kim et al. 2012 [50]	1	1	1	1	0	1	1	1	1	1	1	10
Liao et al. 2017 [51]	1	1	1	1	0	1	0	1	1	1	1	9
Lichtenberg et al. 2019 [52]	1	1	1	1	0	1	1	1	1	1	1	10
Maruya et al. 2016 [53]	1	1	0	1	0	0	0	0	1	1	1	6
Ng et al. 2015 [54]	1	1	1	1	0	0	1	1	1	1	1	9
Park et al. 2017 [55]	1	1	0	1	1	0	0	1	1	1	1	8
Serra-Prat et al. 2017 [56]	1	1	1	1	0	0	0	0	1	1	1	7
Tsekoura et al. 2018 [57]	1	1	1	1	0	0	0	1	1	1	1	8
Vikberg et al. 2019 [58]	1	1	1	1	0	1	1	1	1	1	1	10
Yamada et al. 2019 [59]	1	1	1	1	0	0	1	1	1	1	1	9
Zech et al. 2012 [60]	1	1	1	1	0	0	1	1	1	1	1	9
Zhu et al. 2019 [61]	1	1	1	1	1	1	1	1	1	1	1	11

Scale of item score: O, absent; 1, present. The PEDro scale criteria are: (1) Eligibility criteria specified; (2) Randomized allocation; (3) Concealed allocation; (4) Similarity at baseline on key measures; (5) Blinded participants; (6) Blinded therapists; (7) Blinded assessors; (8) Measure of one key outcome obtained from 85% of participants; (9) Intention-to-treat analysis; (10) Between group statistical comparison of at least one key outcome; (11) Point and variability measures of at least one key outcome.

**Table 2:** Characteristics of the included studies.

Study	Sample (n)	Gender (M/F)	Age (year)	Study Area	Duration (weeks)	Intervention	CG
Aas et al. 2019 [36]	22	7/15	79+	Norway	10	30 min of heavy-load RT training, leg lean mass assessed by DXA, muscle thickness assessed by ultrasound, isometric and dynamic strength, state of torque development and functional capacity, 3 times a week.	Normal daily activities and dietary habits.
Bellomo et al. 2013 [37]	40	10/0	64–80	Italy	12	RT with the intensity 60–80% of maximum force on muscle strength and balance confidence, 10–12 repetitions for 3 sets, twice per week.	Habitual activity level concerning diet, social relations and physical activity. The control group received a minimal intervention consisting of information bulletins with general information about the protocol study and test.
Binder et al. 2002/2005 [38,39]	91 (2002) 115 (2005)	41/50 (2002) 48/67 (2005)	78+	USA	36	(2002): PRT to increase muscle strength and FFM, 1RM, 2–3 times a week. (2005): 22 exercises that focused on flexibility, balance, coordination, speed of reaction, strength, 65% of 1RM, 1 to 2 sets of 6 to 8 repetitions of knee extension, knee flexion, seated bench press, seated row, leg press, biceps curl, after 4 weeks 3 sets of 8 to 12 repetitions performed at 85% to 100% of 1RM, endurance training using treadmills, stationary bicycles, aerodyne bicycles or rowing machines at 65–70% of VO2peak, 3 times per week.	9-month-low-intensity home exercise program, 2–3 times per week. The control intervention consisted of flexibility exercises. Participants in the CG attended a 1-hour training session in exercise facility. To enhance adherence, CG attended a monthly exercise class at exercise facility. They performed the exercises for a 3 three-month interval.
Cadore et al. 2014 [40]	24	7/17	85+	Spain	12	Multicomponent exercise program composed of upper and lower body RT with progressively increased loads (8–10 repetitions, 40–60% of 1RM) with balance and gait retraining, twice per week.	Mobility exercises 30min per day, 4 times per week, small active and passive movements in a form of stretches.
Cebrià i Iranzo et al. 2018 [41]	26	9/17	81+	Spain	12	RT, appendicular skeletal muscle mass (ASM/height^2^, ASM/weight, and ASM/BMI), isometric knee extension, arm flexion and handgrip strength, maximal inspiratory and expiratory pressures, and GV pre- and. postintervention, 3 times a week.	Usual care and daily life activities at the nursing home (lying down, sitting and walking short distances between rooms).
Chan et al. 2012 [42]	117	48/69	65–79	Taiwan	48	RT, dominant leg extension power, 3 times per week.	The educational booklet on frailty, healthy diets, exercise protocols and self-coping strategies-CG was contacted monthly to check on how much they had read it.
Chen et al. 2017 [43]	90	10/80	65–75	Taiwan	12	RT, 60–70% of 1RM, shoulder presses, biceps curls, triceps curls, bench presses, deadlifts, leg swings, squats, standing rows, unilateral rows split front squats, PRT was used every two weeks, 3 sets of 8–12 repetitions with a 2–3 min rest between sets, twice a week.	Day-to-day lifestyle and dietary habits, prohibited from engaging in any exercises.
Chen et al. 2018 [44]	33	0/33	65–75	Taiwan	12	PRT, 8–12 repetitions, upper and lower limb training, kettlebell weight training: kettlebell swing, kettlebell deadlift, kettlebell goblet squat, squat lunge, kettlebell row, single arm kettlebell row, biceps curl, triceps extension, two-arm kettlebell military press, Turkish get up and dynamic workout, 60–70% of 1RM, 60 min, twice a week.	Daily lifestyle without participating in any exercise training.
Clegg et al. 2014 [45]	84	24/60	79	UK	12	RT, TUG to improve mobility and function, 5 days per week.	Usual care from the primary healthcare team.
Fiatarone et al. 1994 [46]	100	37/63	72–98	USA	10	PRT of the hip and knee extensors, 80% of 1-RM, 3 days per week.	Habitual physical activity.
Gené Huguet et al. 2018 [47]	173	62/112	80+	Spain	24	RT program of exercises to gain strength, resistance, balance and coordination, 10 repetitions rising to 15 at two months, 3 times per week.	Standard primary healthcare treatment.
Hassan et al. 2015 [48]	42	no data	78–86	Australia	24	PRT and balance training, lower and upper-body, and the trunk exercises: elbow and shoulder extension (dip), leg press, knee extension and flexion, hip abduction and adduction, abdominal curl and back extension, 2–3 sets per exercise, 10–15 times, increasing the load and repetitions, twice a week.	Usual care, no exercises.
Kim et al. 2011 [49]	155	0/155	75+	Japan	12	60 min of comprehensive physical fitness and muscle mass enhancement training program with PRT, twice per week.	Health education group (once a month for 3 months, a total of three months. The classes focused on cognitive function, osteoporosis and oral hygiene. Regular lifestyle habits and no specific instructions on diet or PA.
Kim et al. 2012 [50]	128	0/128	75+	Japan	12	60 min of strengthening exercises with PRT, stretching, balance and gait training of moderate intensity, twice per week.	Health education group (once a month for 3 months, a total of three months. The classes focused on cognitive function, osteoporosis and oral hygiene. Regular lifestyle habits and no specific instructions on diet or PA.
Liao et al. 2017 [51]	46	0/46	60–80	Taiwan	12	Elastic PRT using Theraband products, 35–40 min, 3 sets and 10 repetitions for each exercise, 3 times per week.	No exercise.
Lichtenberg et al. 2019 [52]	43	43/0	72+	Germany	28	A consistently supervised single-set training on resistance exercise machines using intensifying strategies, underlying physiological parameters, skeletal muscle mass index (SMI), handgrip-strength and gait velocity, twice per week.	Protein supplementation, no exercise.
Maruya et al. 2016 [53]	52	23/29	62–75	Japan	24	Home exercise programs, walking with lower limb RT, body composition, muscle strength and physical performance, 20–30 min per day.	Usual daily activities and exercise for 6 months.
Ng et al. 2015 [54]	246	95/151	65+	Singapore	24	Moderate, gradually increasing intensity, 90 min of duration, included RT integrated with functional tasks and balance training involving functional strength and sensory input, twice a week.	Participants had access to one standard care from health and aged care services that were normally available to older people, including primary and secondary level care from government or private clinics and hospitals, and community-based social, recreational, and day-care rehabilitation services.
Park et al. 2017 [55]	50	0/50	65+	South Korea	24	RT combined with aerobic exercises, 50–80 min, elastic band exercises (elbow flexion, wrist flexion, shoulder flexion, lateral raise, chest press, revere flies, side band, dead lift, squad, leg press, ankle plantar flexion), with progressive repetitions, 5 times per week.	Usual physical activities during 24 weeks, health and family education conducted twice during the intervention period.
Serra-Prat et al. 2017 [56]	172	75/97	70+	Spain	48	30–45 min of aerobic exercises and 20–25 min of RT, strengthening exercises with balance and coordination, 4 times per week.	The usual care and recommendations, no exercise.
Tsekoura et al. 2018 [57]	54	7/47	65+	Greece	24	60 min comprehensive progressive group exercise (RT in a progressive sequence, 20 min of balance and gait training exercises, balance exercises), 2 times per week, walk 30–35 min, 3 times per week.	Educational leaflet about sarcopenia with advice on diet, lifestyle and activity. No exercise.
Vikberg et al. 2019 [58]	70	32/38	70+	Sweden	10	RT programs to increase muscle function and muscle mass using participants’ body weight and suspension bands, 3 times per week.	Usual daily activities, no exercises
Yamada et al. 2019 [59]	112	39/73	65+	Japan	12	30 min of bodyweight RT with slow movement speeds, trunk flexion, hip flexion, hip extension, hip abduction, hip adduction, knee extension and ankle plantar flexion, twice per week.	No exercise.
Zech et al. 2012 [60]	69	no data	65–94	Germany	36	20 min of balance exercises and 25 min of muscle strength and muscle power exercises using RT machines, twice a week.	No exercise.
Zhu et al. 2019 [61]	113	26/87	65+	China	24	90 min group chair-based RT using Thera-bands, and 20 min aerobic exercises, one-home session weekly, gait speed, twice per week.	Usual physical activities and dietary habits during 6-month study period and were subsequently provided with the same exercise program as the IG.

M/F: male/female; 1-RM: one-repetition maximum strength test; 3-RM: tree-repetition maximum strength test; RT: resistance training; PRT: progressive resistance training; MQ: muscle quality; TUG: Timed Up & Go test; POMI: Performance Oriented Mobility Index; FFM: fat-free mass; VO2peak: peak oxygen uptake; GV: gait velocity; ET: endurance training.

**Table 3:** Overall effects of included studies.

Study	Outcome	Measure	Overall Effect
Aas et al. 2019 [36]	Physical performance, Muscle mass, Muscle strength	SPPB, Leg lean mass, Vastus lateralis thickness, Rectus femoris thickness, Vastus intermedius thickness, KE, 1-RM, Habitual GV, five times chair rise, Stair climbing	↑ Leg lean mass (kg); ↓ Fat mass (kg); ↑ Vastus lateralis thickness (% change from baseline); ↑ Knee extension 1-RM; ↑ Rectus femoris thickness (% change from baseline); ↑ Vastus intermedius thickness (% change from baseline); ↑ Five times chair rise (% change from baseline); ↑ Stair climbing (% change from baseline); No significant reduction in habitual GS (% change from baseline).
Bellomo et al. 2013 [37]	Physical performance, Muscle strength	Leg Extension 90º Isometric Test, Sway area, Ellipse Surface, Length of the half-step, Width of the step, Contact Time	↑ Right limb Leg Extension 90º Isometric Test in RT group; ↑ Bilateral limb Leg Extension 90º Isometric Test in RT group; ↓ Open eyes Sway area in RT group; ↓ Closed eyes Sway area in RT group; ↓ Open eyes Ellipse Surface in RT group; Non-significant changes in Closed eyes Ellipse Surface in RT group; ↑ Length of the half-step in RT group; ↑ Width of the step in RT group; ↓ Contact Time in RT group.
Binder et al. 2002/2005 [38,39]	Muscle strength, Body composition	1-RM, Physical Performance Test score, VO2peak, Functional Status Questionnaire score (2002).1-RM, VO2peak, ADL, ET, FSQ, total FFM, PBF, trunk fat, right leg lean mass, right leg fat mass, left leg lean mass, left leg fat mass (2005).	↑ Maximal voluntary force production for knee extension; ↑ Total body FFM in the IG; ↑ Physical Performance Test score in IG and home exercise group; ↑ VO2peak (mL/kg/min) in IG; ↑ Functional Status Questionnaire in IG; ↑ Cybex knee extension 60° (ft/lb) in IG and home exercise group; ↑ Cybex knee flexion 60° (ft/lb) in IG and home exercise group; ↑ Single limb stance time (s) in IG and home exercise group; ↑ Berg Balance Score in IG and home exercise group; Total, trunk, intra-abdominal, and subcutaneous fat mass did not change (2002). ↑ 1.08 ± 11 of change in knee extension 60°/s (ft/lb) in CG; ↑ 5.31 ± 13 of change in knee extension 60°/s (ft/lb) in IG; ↑ 2.11 ± 7 of change in knee extension 60°/s (ft/lb) in CG; ↑ 3.21 ± 8 of change in knee flexion 60°/s (ft/lb) in IG; ↑ 12 ± 10 of change in leg flexion (lb) in IG; ↑ 24 ± 32 of change in leg extension (lb) in IG; ↑23 ± 20 of change in leg press (lb) in IG; ↑ 17 ± 18 of change in seated row in IG; ↑ 95% confidence bounds on the magnitude of improvement in the ET; ↑ 1.0 to 5.2 points for the modified PPT score; ↑ 0.9 to 3.6 mL/kg/min for VO2peak; ↑ 1.6 to 4.9 points for the FSQ score; ↑ 0.0 ± 1.5 of change in total FFM; ↑ −0.4 ± 1.9 of change in PBF (%); ↑ −0.4 ± 1.0 of change in trunk fat (kg); ↑ 0.0 ± 0.3 of change in right leg lean mass (kg); ↑ 0.04 ± 0.2 of change in right leg fat mass (kg); ↓ −0.1 ± 0.4 of change in left leg lean mass (kg); ↑ 0.1 ± 0.3 of change in left leg lean mass (kg) (2005).
Cadore et al. 2014 [40]	Muscle strength, Balance, Physical performance	GS, TUG, raise from a chair, Balance, Falls incidence, HGS, Hip flexion strength, KES, Upper-body 1-RM, Lower-body 1-RM, Maximal power at 30% 1-RM, Maximal power at 60% 1-RM	↓ GS; ↑ TUG (s); ↓ Raise from a chair; ↑ Balance; ↓ Falls incidence; ↑ HGS (N); ↑ Hip flexion strength (N); ↑ KES (N); ↑ Upper-body 1-RM (kg); ↑ Lower-body 1-RM (kg); ↑ Maximal power at 30% 1-RM (W); ↑ Maximal power at 60% 1-RM (W).
Cebrià i Iranzo et al. 2018 [41]	Muscle strength, Muscle mass, Body composition	ASM, Quadriceps femoris strength, Biceps brachii strength, HGS, MIP, MEP, MVV, GS	No change in ASM (kg/m2); No change in ASM/BMI (m2); ↑ Quadriceps femoris strength (kg) in RMTG and PMTG; ↑ Biceps brachii strength in PMTG; ↓ Biceps brachii strength in RMTG; ↑ HGS D in RMTG and PMTG; ↑ MIP D in RMTG and PMTG; ↑ MEP in RMTG and PMTG; ↑ MVV in RMTG and PMTG; ↑ GS in RMTG; No change in Gate speed in PMTG.
Chan et al. 2012 [42]	Muscle strength, Body composition	Dominant leg extension power, left OLS, FFM, BMI, one leg stand time	↑ Dominant leg extension power; ↑ Vitamin D level (4.9 ± 7.7); ↓ Osteopenia (74%); ↑ −0.31 ± 1.19 of change in BMI (kg/m2) in IG; ↑ −0.46 ± 1.36 of change in FFM (kg) in IG; ↑ 3.69 ± 9.15 of change in left one leg stand time (s) in IG; ↓ −6.44 ± 10.08 of change in dominant leg extension power (kg) in IG.
Chen et al. 2017 [43]	Body composition, Muscle strength, Muscle mass	Weight, SMM, ASM/weight, BFM, BMI, PBF, VFA, HGS, back extensor, KES	↑ Weight (kg) in CG; ↓ Weight (kg) in IG; ↑ SMM (kg) in IG; ↓ SMM (kg) in CG; ↓ ASM/weight (%) in CG; ↑ ASM/weight (%) in IG; ↓ BFM (kg) in IG; ↑ BFM (kg) in CG; ↓ BMI (kg/m2) in IG; ↑ BMI (kg/m2) in CG; ↓ PBF (%) in IG; ↑ PBF (%) in CG; ↓ VFA (cm2) in IG; ↑ VFA (cm2) in CG; ↑ HGS (kg) in IG; ↓ HGS (kg) in CG; ↑ Back extensor (kg) in IG; ↓ Back extensor (kg) in CG; ↑ KES (kg) in IG; ↓ KES (kg) in CG.
Chen et al. 2018 [44]	Body composition, Muscle mass, Muscle strength	Weight, SMM, BFM, VFA, ASM, left HGS, right HGS, BS	↓ Weight (kg) in IG; ↑ Weight (kg) in CG; ↑ SMM (kg) in IG; ↓ SMM (kg) in CG; ↓ BFM (kg) in IG; ↑ BFM (kg) in CG; ↓ VFA (cm2) in IG; ↑ VFA (cm2) in CG; ↑ ASM (kg) in IG; ↓ ASM (kg) in CG; ↑ left HGS (kg) in IG; ↑ left HGS (kg) in CG; ↑ right HGS (kg) in IG; ↓ right HGS (kg) in CG; ↑ BS in IG; ↓ BS in CG.
Clegg et al. 2014 [45]	Muscle strength, Physical performance	TUG	↑ −10.4 ± 64.0 of change in TUG (s) in IG; ↑ −39.1 ± 90.6 of change in TUG (s) in CG.
Fiatarone et al. 1994 [46]	Muscle strength, Body composition	GV, SCPT, CSA, weight, thigh-muscle area (cm2)	↑ GV (11.8 ± 3.8%); ↑ Right knee strength (kg) (4.9 ± 0.6%) in IG; ↑ Left knee strength (kg) (5.2 ± 0.6%) in IG; ↑ Right hip (kg) (8.8 ± 1.2%) in IG; ↑ Left hip (kg) (8.1 ± 1.0%) in IG; ↑ Right leg press (kg) (8.3 ± 2.9%) in IG; ↑ Left leg press (kg) (9.3 ± 2.1%) in IG; ↑ Weight (kg) (0.2 ± 0.4%) in IG; ↑Thigh-muscle area (cm2) (0.9 ± 1.7%) in IG; ↑ SCPT (28.4 ± 6.6%); ↑ CSA (2.7 ± 1.8%) in IG; ↓ CSA (1.8 ± 2.0%) in CG.
Gené Huguet et al. 2018 [47]	Muscle strength, Physical performance	TUG, FTSST	↓ Frailty (95%CI); ↑ Reversion to robustness (14.1%); ↑ Quality of life; ↑ Functional mobility (FTSST) in CG and IG; ↑ TUG in IG.
Hassan et al. 2015 [48]	Body composition, Muscle strength, Muscle mass, Physical performance	Weight, BF, BMI, SMMI, Lean mass, HGS, GS	↓ Weight (kg) in IG; ↓ Weight (kg) in CG; ↓ BF (%) in IG; ↑ BF (%) in CG; ↓ BMI (kg/m2) in IG; ↓ BMI (kg/m2) in CG; No significant difference in SMMI (kg/m2) in IG; ↓ SMMI (kg/m2) in CG; ↑ Lean mass (kg) in IG; ↓ Lean mass (kg) in CG; ↑ HGS (kg) in IG; ↓ HGS (kg) in CG; ↑ GS (m/s) in IG; ↓ GS (m/s) in CG.
Kim et al. 2011 [49]	Muscle strength, Physical performance, Muscle mass	Leg muscle mass, Usual WS, KES	↑ Legs muscle mass (kg) in exercise group, home exercise group and exercise + amino acid supplementation group; ↑ Usual WS (m/s) in exercise group, home exercise group, exercise + amino acid supplementation group and amino acid supplementation group; ↑ KES (Nm/kg) in exercise group and exercise + amino acid supplementation group; ↓ Maximal walking speed (m/s) in exercise group, home exercise group, exercise + amino acid supplementation group and amino acid supplementation group; ↑ Appendicular muscle mass (kg) in exercise group, exercise + amino acid supplementation group and amino acid supplementation group; ↑ Muscle mass (kg) in exercise group, home exercise group, exercise + amino acid supplementation group and amino acid supplementation group.
Kim et al. 2012 [50]	Muscle strength, Physical performance, Muscle mass	Muscle mass, Legs muscle mass, ASM, HGS, Usual walking speed, Maximum walking speed, TUG, KES	↓ Muscle mass (kg) in home exercise group; ↑ Legs muscle mass (kg) in exercise group, home exercise group and exercise + tea catechin supplementation group; ↓ ASM (kg) in home exercise group; ↑ Grip strength (kg) in exercise group, exercise + tea catechin supplementation group and tea catechin supplementation group; ↑ Usual walking speed (m/s) in exercise group and exercise + tea catechin supplementation group; ↑ Maximum walking speed (m/s) in exercise group and exercise + tea catechin supplementation group;↑ TUG (s) in exercise group, exercise + tea catechin supplementation group and tea catechin supplementation group; ↓ KES(Nm) in exercise group, home exercise group, exercise + tea catechin supplementation group and tea catechin supplementation group.
Liao et al. 2017 [51]	Body composition, Muscle strength, Physical performance, Muscle masss, Muscle quality	FFM, LLM, TFM, PBF, SLS, GS, TUG, TCR, HGS, LE, UE, LE	↑ FFM (kg) in IG; ↓ FFM (kg) in CG; ↑ LLM (kg) in IG; ↓ LLM (kg) in CG; ↓ TFM (kg) in IG; ↑ TFM (kg) in CG; ↓ PBF (%) in IG; ↑ PBF (%) in CG; ↑ SLS (s) in IG; ↓ SLS (s) in CG; ↑ GS (m/s) in IG; ↓ GS (m/s) in CG; ↑ TUG (s) in IG;↓ TUG (s) in CG; ↑ TCR (rep) in IG; ↑ TCR (rep) in CG; ↑ HGS (kg) in IG; ↑ HGS (kg) in CG; ↑ LE (N) in IG; ↓ LE (N) in CG; ↑ UE (kg/kg) in IG; ↓ UE (kg/kg) in CG; ↑ LE (N/kg) in IG; ↓ LE (N/kg) in CG.
Lichtenberg et al. 2019 [52]	Muscle mass, Muscle strength, Physical performance	Muscle mass, Habitual GV, HGS	↑ Skeletal muscle mass index (SMI) (kg/m2) in HI-RT; Skeletal muscle mass index (SMI) (kg/m2) in CG; ↑ Habitual GV (m/s) in HI-RT; ↓ Habitual GV (m/s) in CG); ↑ HGS (kg) in HI-RT; ↓ HGS (kg) in CG).
Maruya et al. 2016 [53]	Body composition, Muscle strength Physical performance	SMMI, HGS, WS, KES, SLS,%BF	↑ SMI (kg/m2) in IG; ↑ HGS (kg) in IG; ↓%BF (%) in IG; ↓ WS (comfortable) (m/s) in IG; ↑ WS (maximum) (m/s) in IG; ↑ KES (Nm/kg) in IG;↑ SLS (s) in IG.
Ng et al. 2015 [54]	Body composition, Muscle strength, Physical performance	BMI, KES, GS, Physical activity, Energy, IADL-ADL dependency	↓ BMI (mean change −0.01 in IG); ↑ KES (kg) (mean change 1.83 in IG); ↑ KES (kg) (mean change 1.13 in CG); ↑ GS (s) (mean change −1.29 in IG and −0.56 in CG); ↑ Physical activity (mean change 23.2 in IG and 8.02 in CG); ↑ Energy (mean change 0.96 in IG and 0.59 in CG); ↑ IADL-ADL dependency (%).
Park et al. 2017 [55]	Body composition, Muscle strength, Muscle mass, Physical performance	PBF, waist circumference, ASM, left HGS, right HGS, Chair stand test, sit and reach, MWS, two-minute step	↓ PBF (%) in IG; ↑ PBF (%) in CG; ↓ Waist circumference (cm) in IG; ↑ Waist circumference (cm) in CG; ↑ ASM (kg) in IG; ↓ ASM (kg) in CG; ↑ Left HGS (kg) in IG; ↓ Left HGS (kg) in CG; ↑ Right HGS (kg) in IG; ↓ Right HGS (kg) in CG; ↑ Chair stand test (rep/30s) in IG; ↓ Char stand test (rep/30s) in CG; ↑ Sit and reach (cm) in IG; ↓ Sit and reach (cm) in CG; ↑ MSW (m/s) in IG; ↓ MSW (m/s) in CG; ↑ Two-minute step (rep) in IG; ↓ Two-minute step (rep) in CG.
Serra-Prat et al. 2017 [56]	Body composition, Muscle strength, Physical performance	BMI, GS, TUG, HGS, WS	↑ BMI in women 0,05 (−0,66 to 0,75); ↓ BMI in men −0,46 (−1,20 to 0,27); ↑ Outdoor walking (h/day) (1.0 ± 0.6) in IG; ↑ WS (m/s) (1.0 ± 0.2) in IG; ↑ GS (m/s) −0,35 (-0,77 to 0,08); ↑ TUG (s) −0,04 (−0,64 to 0,57);↑ HGS (kg) in men 1,17 (−0,95 to 3,29); ↓ HGS (kg) in women −0,58 (−2,41 to 1,26).
Tsekoura et al. 2018 [57]	Body composition, Muscle mass, Muscle strength, Physical performance	BMI, SMMI, TUG, 4 m test, GS, Chair stand test, HGS, FFM, Calf circumference, Isokinetic measurements	↓ BMI (kg/m2) in IG; ↑ BMI (kg/m2) in HE; ↑ FFM in IG; ↓ FFM in HE; ↑ SMI (kg/m2) in IG; ↑ SMI (kg/m2) in HE; ↑ Calf circumference (cm) in IG; ↑ Calf circumference (cm) in IG; ↑ TUG (s) in IG; ↑ TUG (s) in HE; ↑ 4 m test (s) in IG; ↑ 4 m test (s) in HE; ↑ GS (m/s2) in IG; ↑ GS (m/s2) in HE; ↓ Chair stand test (s) in IG; ↓ Chair stand test (s) in HE; ↑ HGS (kg) in IG; ↑ HGS (kg) in HE; ↑ Right knee extension 90 (Nm/kg) in IG; ↑ Right knee extension 90 (Nm/kg) in HE; ↑ Right knee extension 180 (Nm/kg) in IG; ↓ Right knee extension 180 (Nm/kg) in HE; ↑ Right knee flexion 90 (Nm/kg) in IG; ↑ Right knee flexion 90 (Nm/kg) in HE; ↑ Right knee flexion 180 (Nm/kg) in IG; ↑ Right knee flexion 180 (Nm/kg) in HE; ↑ Left knee extension 90 (Nm/kg) in IG; ↑ Left knee extension 90 (Nm/kg) in HE; ↑ Left knee extension 180 (Nm/kg) in IG; ↑ Left knee extension 180 (Nm/kg) in HE; ↑ Left knee flexion 90 (Nm/kg) in IG; ↑ Left knee flexion 90 (Nm/kg) in HE; ↑ Left knee flexion 180 (Nm/kg) in IG; ↑ Left knee flexion 180 (Nm/kg) in HE.
Vikberg et al. 2019 [58]	Muscle strength, Muscle mass, Body composition, Physical performance	SPBB, TUG, HGS, DXA measurement	↑ Walk; ↑ Sit to stand; No change in balance; ↑ TUG; ↑ Handgrip strength; ↓ Total fat mass (kg); ↑ Total lean mass (kg); ↑ Arm lean mass (kg); ↑ Leg lean mass (kg); ↑ ALMI.
Yamada et al. 2019 [59]	Muscle mass, Physical performance	Knee extension, ASM, Echo intensity for rectus femoris, Echo intensity for vastus intermedius, Comfortable walking time, Maximum walking time, OLS, Five chair stands time, HGS	↑ Knee extension (Nm) (6.76 ± 11.04) in exercise + nutrition group and (0.85 ± 8.98) in exercise group; ↑ ASM (0.07 ± 1.11) in exercise group; Non-significant change in ASM (0.00 ± 1.29) in exercise + nutrition group; ↑ Echo intensity for rectus femoris (−8.47 ± 13.20) in exercise + nutrition group and (5.29 ± 14.12) in exercise group; ↑ Echo intensity for vastus intermedius (1.37 ± 14.13) in exercise + nutrition group and (5.86 ± 13.34) in exercise group; ↓ Comfortable walking time (s) (−0.82 ± 1.52) in exercise + nutrition group and (−0.90 ± 2.31) in exercise group; ↓ Maximum walking time (s) (−0.72 ± 1.12) in exercise + nutrition group and (−0.30 ± 1.76) in exercise group; ↑ OLS (s) (1.43 ± 4.64) in exercise + nutrition group and (0.60 ± 10.10) in exercise group; ↓ Five chair stands time (s) (−1.63 ± 3.69) in exercise + nutrition group, (−0.87 ± 3.95) in exercise group; ↓ HGS (kg) in exercise group (–0.05 ± 2.27); ↑ HGS (kg) in exercise + nutrition group (0.77 ± 1.80).
Zech et al. 2012 [60]	Body composition, Muscle strength, Physical performance	BMI, Mass, SPPB, Balance, GS, Chair rise, ALM, SF-LLFDI, Power	↓ BMI (kg/m2) 28.7 ± 4.1 in IG; ↑ BMI (kg/m2) 28.5 ± 4.0 in CG; ↓ Mass (kg) 78.0 ± 10.0 in IG and 75.8 ± 13.5 in CG; ↑ SPPB (pt) 9.7 ± 2.2 in IG; ↓ SPPB (pt) 9.7 ± 2.1 in CG; ↑ Balance (pt) 2.8 ± 1.3 in IG; ↓ Balance (pt) 2.8 ± 1.1 in CG; ↑ GS (pt) 3.7 ± 0.6 in IG; ↑ GS (pt) 3.7 ± 0.6 in CG; ↑ Chair rise (pt) 3.3 ± 1.0 in IG; ↑ Chair rise (pt) 3.1 ± 1.2 in CG; ↑ aLM (kg) 18.0 ± 3.3 in IG and 17.5 ± 2.6 in CG; ↑ SF-LLFDI (pt) in IG; ↓ SF-LLFDI (pt) in CG; ↓ Power (W) in IG and CG.
Zhu et al. 2019 [61]	Body composition, Muscle strength, Physical performance	GS, Upper limb fat mass, Lower limb fat mass, Upper limb muscle mass, Lower limb muscle mass, ASM, MGS, Leg extension, Medicine ball, Five-chair stand, 6MWD	↑ GS (m/s) in exercise group; ↑ Upper limb fat mass (kg) in exercise group; ↓ Lower limb fat mass (kg) in exercise group; ↑ Upper limb muscle mass (kg) in exercise group; ↑ Lower limb muscle mass (kg) in exercise group; ↑ ASM (kg/m2) in exercise group; ↑ MGS (kg) in exercise group; ↑ Leg extension (kg) in exercise group; ↑ Medicine ball (m) in exercise group; ↓ Five-chair stand (s) in exercise group; ↑ 6MWD (m) in exercise group.

CG: control group; IG: intervention group; RT: resistance training; HGS: hand grip strength; TUG: Time Up and Go test; SPPB: short physical performance battery; 1-RM: 1-repetition maximum strength test; IMS: isometric muscle strength; HHD: hydraulic hand dynamometer (Jamar; Patterson Medical, Warrenville, IL); ID: isokinetic dynamometer (Cybex Norm, Cybex International Inc., NY, USA); IS: isokinetic strength (CYBEX 330 System); BFM: body fat mass; FFM: fat free mass; ET: intensive exercise training; VO2peak: peak oxygen uptake; ADL: activity of daily living; BMI: body mass index; FSQ: Functional Status Questionnaire; modified PPT: Modified Physical Performance Test; ASM: Appendicular skeletal muscle mass; KE: knee extension; MSIP: maximum static inspiratory pressure; MSEP: maximum static expiratory pressure; CSA: Cross-sectional area; OLS: One Leg Stand test; 5MWT: Five-Minute Walk Test; GV: gait velocity; SCPT: Stair Climbing Power Test; FTSST: Five Time Sit To Stand Test; 10MWT: 10-Meter Walk Test; SRT: Sitting-Rising Test; GV: Gait Velocity; PA: physical activity; HRQL: Health-related quality of life; IADL: Instrumental activities of daily living; 6MWD: 6-min walking distance; ALMI: Appendicular lean mass index; ALM: Appendicular lean mass; MIP: Maximal Inspiratory Pressure; MEP: Maximal Expiratory Pressure; MVV: Maximal Voluntary Ventilation; PMTG: Peripheral Muscle Training Group; RMTG: Respiratory Muscle Training Group; D: Dominant side; pKEMP-dBW: Percent knee extension muscle power to dry body weight; TLM: Total Lean Mass; MQ: Muscle Quality; HBRE: Home-Based Resistance Exercise; BMD: Bone Mineral Density; HI-RT: High-Intensity Resistance Training Group; SMI: Skeletal muscle index; WS: walking speed; SLS: Single leg standing; STS: 30-s sit-to-stand; IKE: Isometric knee extension; GS: Gait speed; SMM: Skeletal muscle mass; SMMI: Skeletal muscle mass index; CE: Closed eyes; OE: Open eyes; HE: Home-based exercise; MGS: Maximum grip strength; SF-LLFDI: Short form of the Late Life Function and Disability Instrument; ADL: Activities of daily living; IADL: instrumental activities of daily living; PBF: percent body fat; VFA: visceral fat area; BS: back strength; BF: body fat (%); LLM: lean leg mass; TFM: total fat mass; TCR: timed chair rise; LE: lower extremity; UE: upper extremity; MWS: maximum walking speed.

## Data Availability

The data presented in this study are available on request from the corresponding author.

## Appendix A

**Table A1:** The search terms used in the review to identify resistance exercise intervention designed to improve (pre-)sarcopenic and (pre-)frail older adults’ strength, physical function and body composition. These search terms were used in PubMed, Web of Science, Cochrane Library and Scopus databases. **PubMed Filters:** Randomized controlled trial, Full text, 1975–2020, Aged 65+, Humans, English.

Variable	Terms
Outcome term	(“sarcopenia” OR “presarcopenia” OR “*sarcopenia” OR “pre-sarcopenia” OR “presarcopenic” OR “pre-sarcopenic” OR “sarcopenic” AND “frailty” OR “*frailty” OR “prefrailty” OR “pre-frailty” OR “frail” OR “frail*” OR “prefrail” OR “pre-frail”)
Measurement parameters	(“muscle strength” OR “muscular strength” OR “muscle mass” OR “fat mass” OR “FFM” OR “body composition” OR “gait speed” OR “gait” OR “walking speed” OR “balance” OR “appendicular skeletal muscle mass” OR “body fat mass” OR “balance test” OR “HGS” OR “hand strength” OR “grip strength” OR “lower limb strength” OR “chair stand time” OR “knee extension 1-RM” OR “TUG” OR “agility” OR “SPPB” OR “physical function” OR “one leg stand time” OR “body fat percentage” OR “total body fat mass” OR “upper limb fat mass” OR “lower limb fat mass” OR “walking speed” OR “knee flexion” OR “knee extension isokinetic” OR “knee extension isometric” OR “leg lean mass” OR “thigh muscle area” OR “muscle mass” OR “fat mass” OR “fat-free mass” OR “calf circumference” OR “arm lean mass” OR “total lean mass” OR “appendicular lean mass” OR “upper limb muscle mass” OR “lower limb muscle mass”)
Exercise intervention	(“resistance training” OR “resistance exercise” OR “resistance exercises” OR “resistance” OR “strength training” OR “strength exercise” OR “strength exercises” OR “physical strength” OR “physical activity” OR “strength” OR “physical strength” OR “weight training” OR “weight” OR “exercises” OR “exercise” OR “training” OR “physical activity” OR “physical activities” OR “physical training” OR “physical fitness” OR “weight exercises” OR “weight exercise” OR “weight-bearing training” OR “weight-bearing exercises” OR “weight bearing exercise” OR “weight bearing training”)
Study design	(“randomized controlled trial*” OR “controlled” OR “RCT” OR “clinical trial” OR “controlled clinical trial” OR “randomized controlled trials” OR “random allocation” OR “double blind method” OR “single blind method” OR “clinical trials” OR “single” OR “double” OR “triple” OR “placebos” OR “research design” OR “follow-up stud*” OR “placebo” OR “random” OR “comparative study” OR “evaluation studies” OR “prospective stud*” OR “control” OR “prospective*” OR “volunteer” OR “research design” OR “control”)
Population	(“old” OR “age” OR “old age” OR “older adult*” OR “older people” OR “elderly” OR “elder*” OR “people” OR “aging adults” OR “ageing adults” OR “geriatric*” OR “senile people” OR “senile person” OR “older person*” OR “old-age” OR “older” OR “person*” OR “adult*” OR “elderly person*” OR “senior*” OR “aging” OR “ageing” OR “aged” OR “old man” OR “old men” OR “old woman” OR “old women” OR “older woman” OR “older women”)

**Table A2:** Diagnostic criteria for frailty or sarcopenia and prevalence of participants for each study.

Study	Nr of Participants	Gender (M*/F*)	Age, year	Study Area	Duration (w)	Diagnostic Criteria	% of Participants with Sarcopenia/Frailty
Aas et al. 2019 [36]	22	7/15	79+	Norway	10	SPPB for functional capacity-score of 10 or less out of 12 (timed standing valance, GS, TUG)	100% of older adults with frailty
Bellomo et al. 2013 [37]	40	10/0	64–80	Italy	12	Criteria of the CDCP-sarcopenia defined as a muscle mass index (muscle mass [kg]/height m^2^) less than two SD below the mean of a young reference population	100% of older adults with sarcopenia
Binder et al. 2002/2005 [38,39]	91 (2002) 115 (2005)	41/50 (2002) 48/67 (2005)	78+	USA	36	Measures with established predictive validity for disability and mortality in older adults-at least two out of three frailty criteria: 1. Score between 18 and 31 on the modified PPT, 2. Report of difficulty or need for assistance with up to two IADLs or one IADL, 3. Achievement of a VO2 peak between 10 to 18 mL · kg^−1^ · min^−1^ (Binder 2002) 2 of three of the criteria: 1. modified PPT score between 18 and 32 (maximum score 36), 2. Report on difficulty and/or assistance with up to two IADLs and/or one IADL, 3. Peak aerobic power (VO2peak) between 10- and 18 mL kg^−1^ · min^−1^ (Binder 2005)	100% of older adults with mild to moderate frailty
Cadore et al. 2014 [40]	24	7/17	85+	Spain	12	Fried’s criteria for frailty-presence of three or more of the following components: slowness, weakness, weight loss, exhaustion, low physical activity	100% of older adults with frailty
Cebrià i Iranzo et al. 2018 [41]	26	9/17	81+	Spain	12	Compliance of the sarcopenia diagnostic criteria proposed by Tyrovolas et al. 2015 which include: 1. Skeletal Muscle Mass Index (SMI = Appendicular Skeletal Muscle Mass/Body Mass Index) with cut-off points for Spanish population (≤0.93 for male and ≤0.57 for female), 2. Gait speed with cut-off points according to sex, height and age (between 0.95–0.66 m/s for male and 0.80–0.48 m/s for female)	100% of older adults with sarcopenia
Chan et al. 2012 [42]	117	48/69	65–79	Taiwan	48	CCSHA_CFS_TV with satisfactory inter-rater reliability and criterion validity was used for the first stage screening. Eligible participants scored 3–6 on the CCSHA_CFS_TV (scores 1,2-too healthy or 7-too ill)	100% of older adults with frailty
Chen et al. 2017 [43]	90	10/80	65–75	Taiwan	12	Sarcopenia defined as ASM [kg]/weight [kg] × 100%	100% of older adults with sarcopenia
Chen et al. 2018 [44]	33	0/33	65–75	Taiwan	12	Asian Working Group for Sarcopenia criteria-the sarcopenic cut-off value for muscle mass measurement is <5.7 kg/m^2^ for women, with ASM serving as a sarcopenia index (defined as ASM/height [kg/m^2^] analysed using bioelectrical impedance analysis. Muscle strength-cut-off value for HGS was set as <18 kg for women when HGS was used as a sarcopenia index	100% of older adults with sarcopenia
Clegg et al. 2014 [45]	84	24/60	79	UK	12	To account for the spectrum of frailty, the HOPE programme was graded into three levels: 1. TUG as a basic mobility test with good accuracy for identifying frailty (≥30 s level 1), 2. TUG in 20–29 s, intermediate level, 3. TUG in <20 s, independently mobile older adults	100% of older adults with frailty
Fiatarone et al. 1994 [46]	100	37/63	72–98	USA	10	Boston FICSIT	100% of older adults with frailty
Gené Huguet et al. 2018 [47]	173	62/112	80+	Spain	24	Fried’s criteria for pre-frailty (slowness, weakness, weight loss, exhaustion, low physical activity), Comprehensive Geriatric Assessment (VGI)-VGI-Frail, inter-RAI frailty scale, the Clinical Frailty Scale for frailty	86.5% of older adults with pre-frailty (13.5% did not finish the training program)
Hassan et al. 2015 [48]	42	no data	78–86	Australia	24	EWGSOP criteria-low muscle mass and low muscle function (muscle strength or physical performance)	100% of older adults with sarcopenia
Kim et al. 2011 [49]	155	0/155	75+	Japan	12	ASM/height 2 less than 6.42 kg/m^2^, knee extension strength less than 1.01 Nm/kg, BMI less than 22.0 kg/m	100% of older adults with sarcopenia
Kim et al. 2012 [50]	128	0/128	75+	Japan	12	ASM/height 2 less than 6.42 kg/m^2^, knee extension strength less than 1.01 Nm/kg, BMI less than 22.0 kg/m	100% of older adults with sarcopenia
Liao et al. 2017 [51]	46	0/46	60–80	Taiwan	12	EWGSOP criteria: low muscle mass-pre-sarcopenia, low muscle mass and/or low physical performance-sarcopenia	100% of older adults with sarcopenia
Lichtenberg et al. 2019 [52]	43	43/0	72+	Germany	28	FrOST (SMI <7.50 kg/m^2^)	100% of older adults with sarcopenia
Maruya et al. 2016 [53]	52	23/29	62–75	Japan	24	AWGS criteria, pre-sarcopenia: SMI <7.0 kg/m^2^ for men and <5.7 kg/m^2^ for women, sarcopenia: HGS <26 kg for men and < 8 kg for women	85% of older adults with pre-sarcopenia 15% of older adults with sarcopenia
Ng et al. 2015 [54]	246	95/151	65+	Singapore	24	5 CHS criteria for frailty: unintentional weight loss (<18.5 kg/m^2^ or self-reported unintentional weight loss ≥4.5 kg), slowness, weakness, exhaustion and low activity (1 if present and 0 if absent)	72% of older adults with pre-frailty 28% of older adults with frailty
Park et al. 2017 [55]	50	0/50	65+	South Korea	24	BMI ≥25.0 kg/m^2^, ASM/weight <25.1%	100% of older adults with sarcopenia
Serra-Prat et al. 2017 [56]	172	75/97	70+	Spain	48	Fried’s criteria for frailty-presence of three or more of the following components: slowness, weakness, weight loss, exhaustion, low physical activity	100% of older adults with pre-frailty
Tsekoura et al. 2018 [57]	54	7/47	65+	Greece	24	SarQol_GR (22 questions, rated on a 4-point Likert scale of frequency and intensity): physical and mental health, locomotion, body composition, functionality, ADL, leisure activities and fears (0-worst imaginable health, 100-best imaginable health), EWGSOP	100% of older adults with sarcopenia
Vikberg et al. 2019 [58]	70	32/38	70+	Sweden	10	EWGSOP criteria: ALMI (arm lean mass + leg lean mass divided by height squared) ≤7.29 (range 5.69–7.29) in men and ≤5.93 (range 4.50–5.93) in women	100% of older adults with pre-sarcopenia
Yamada et al. 2019 [59]	112	39/73	65+	Japan	12	AWGS criteria, low muscle function (low physical performance or low muscle strength) and low muscle mass	30% of older adults with sarcopenia (70% of older adults with dynapenia, not included in our meta-analysis)
Zech et al. 2012 [60]	69	no data	65–94	Germany	36	Fried’s criteria for frailty-presence of three or more of the following components: slowness, weakness, weight loss, exhaustion, low physical activity (Minnesota Leisure Time Physical Activity Questionnaire)	100% of older adults with pre-frailty
Zhu et al. 2019 [61]	113	26/87	65+	China	24	AWGS criteria: ASM/height 2(ASM/Ht2) measured using DXA of less than 7.0 kg/m^2^ for men and 5.4 kg/m^2^ for women; low HGS (less than 26 kg for men and 18 kg for women) and/or low usual GS (less than or equal to 0.8 m/s)	100% of older adults with sarcopenia

M/F: male/female; SD: standard deviation; CDCP: Centers for Disease Control and Prevention; PPT: Physical Performance Test; VO2 peak: measurement of peak oxygen uptake; IADL: instrumental activities of daily living; basic ADLs: basic activities of daily living; CCSHA_CFS_TV: The Chinese Canadian Study of Health and Aging Clinical Frailty Scale Telephone Version; ASM: appendicular skeletal muscle mass; HGS: handgrp strength; TUG: Time Up and Go; HOPE: the Home-based Older People’s Exercise; FICSIT: Frailty and Injuries: Cooperative Studies of Intervention Techniques; EWGSOP: the European Working Group on Sarcopenia in Older People; BMI: body mass index; FrOST: Franconian Sarcopenic Obesity Study; SMI: skeletal muscle mass index; AWGS: Asian Working Group for Sarcopenia; ADL: Activities of Daily Living; DXA: Dual Energy X-ray Absorptiometry; GS: Gait Speed.
